# A platform for the rapid screening of equine immunoglobins F (ab)2 derived from single equine memory B cells able to cross-neutralize to influenza virus

**DOI:** 10.1080/22221751.2024.2396864

**Published:** 2024-09-27

**Authors:** Yuezhi Lin, Yayu Wang, Hongxin Li, Tong Liu, Jiaqi Zhang, Xing Guo, Wei Guo, Yaoxin Wang, Xiangning Liu, Shaoli Huang, Huaxin Liao, XiaoJun Wang

**Affiliations:** a State Key Laboratory for Animal Disease Control and Prevention, Harbin Veterinary Research Institute of Chinese Academy of Agricultural Sciences, Harbin, People’s Republic of China; b Zhuhai Trinomab Pharmaceutical Co., Ltd, Zhuhai, People’s Republic of China; c Institute of Western Agriculture, the Chinese Academy of Agricultural sciences, Changji, People’s Republic of China; d Clinical Research Platform for Interdiscipline of Stomatology, The First Affiliated Hospital of Jinan University, Guangzhou, People's Republic of China; e Department of Stomatology, College of Stomatology, Jinan University, Guangzhou, People’s Republic of China; f The Hong Kong University of Science and Technology, School of Engineering, Hong Kong, People’s Republic of China

**Keywords:** Single B cells-based antibody platform, neutralizing antibodies, equine immunoglobulins F(ab)2, influenza viruses, vaccine design

## Abstract

Single B cells-based antibody platforms offer an effective approach for the discovery of useful antibodies for therapeutic or research purposes. Here we present a method for screening equine immunoglobins F(ab)2, which offers the potential advantage of reacting with multiple epitopes on the virus. Using equine influenza virus (EIV) as model, a hemagglutinin (HA) trimer was constructed to bait B cells in vaccinated horses. We screened 370 HA-specific B cells from 1 × 10^6^ PBMCs and identified a diverse set of equine variable region gene sequences of heavy and light chains and then recombined with humanized Ig Fc. Recombinant equine Ig was then self-assembled in co-transfected 293 T cells, and subsequently optimized to obtain HA binding B-cell receptor (s). The recombinant antibodies exhibited a high binding affinity to the HA protein. Antibody H81 exhibited the highest cross neutralizing activities against EIV strains *in vitro*. Furthermore, it effectively protected EIV-challenged mice, resulting in significantly improved survival, reduced pulmonary inflammation and decreased viral titers. *In silico* predication identified a functional region of H81 comprising 27 key amino acids cross the main circulating EIV strains. The 12 amino acid residues in this region with the highest binding affinities were screened. Notably, the predicted epitopes of H81 encompassed the documented equine HA receptor binding site, validating its cross-neutralization. In summary, a rapid platform was successfully established to investigate the profiling of equine antigen-recognizing receptors (BCRs) following infection. This platform has the potential to optimize the screening of virus-neutralizing antibodies and aid in vaccine design.

## Introduction

Viral infectious diseases pose a significant and persistent threat to public health, even as numerous vaccines against different viruses have been developed, administered and boosted. This persistence is primarily attributed to the emergence of novel virus variants and the waning immunity responses to vaccines [1–4]. In recent years, mounting evidence has suggested that broadly neutralizing antibodies (bNAbs) might have great promise as antiviral agents or vaccine adjuvants for the prevention and treatment of viral infections [5–7]. Therefore, there is an urgent need to screen neutralizing antibodies elicited by virus infection or vaccines to obtain cross-neutralizing antibodies.

Immunoglobulin G has been widely utilized for screening monoclonal antibodies (mAbs) coding Fragment antigen binding (Fab) regions, which contain the B-cell antigen-recognizing receptor (BCR) domain. Better understanding of the anti-viral function of the BCR would greatly facilitate the development of potent neutralizing mAbs and promote the design of efficient vaccines. Single B-cell technologies enable the rapid and efficient retrieval of naturally paired heavy and light chain repertoires from individual cells [8–10]. Therefore, they are powerful tools for screening antibodies with high neutralization, and might also rapidly decipher the molecular scenario between the emerging virus and host B cell immunity with correspondingly rearranged BCRs [8,11]. Multiple studies have shown that the equine immunoglobulins F(ab)2 have the potential advantage of reacting with multiple epitopes on viral proteins, reducing the likelihood of therapy resistance driven by mutations [12,13]. Thus, horses might serve as important reactors for production of heterologous antibodies [14]. However, there is still a lack of effective, reproducible methods for rapidly screening equine F(ab)2 with neutralization activity against virus infection, especially when dealing with a rapidly mutating virus.

Equine influenza virus is a prototypical example of a virus characterized by rapid antigenic mutation [15,16]. Currently, two subtypes of equine influenza virus are recognized; H7N7 and H3N8. Although the last confirmed report of H7N7 in horses was in 1979, H3N8 viruses have been detected worldwide since 1963 [16]. Although hemagglutinin (HA) is the primary target for antibody production, it undergoes continuous change (antigenic drift) or is genetically reassorted (antigenic shift) to escape host immunity. From an evolutionary standpoint, it is intriguing that while the receptor binding site of HA head triggers robust host B-cell immunity, it simultaneously facilitates immune evasion by constantly evolving to evade antibodies targeting it, thus perpetuating antigenic drift and shift. To better understand this, we propose a platform utilizing EIV-HA as an antigenic model to refine cross-reactive equine monoclonal immunoglobulins from single B cells within equine BCR reservoirs. Our objective is to establish an efficient cell-based method for the rapid generation of equine immunoglobulins F(ab)2. Ultimately, this approach aims to facilitate the selection of neutralizing antibodies against the virus, providing valuable insights to expedite the development of universal vaccine designs.

## Materials and methods

### Ethics statement

The mice employed in the mouse experiments were kept in the animal BSL-2 facility at the Harbin Veterinary Research Institute (HVRI), the Chinese Academy of Agricultural Sciences (CAAS), where the animal experiments were conducted. Mice were purchased from Liao-Ning Changsheng Biotechnology. All animal experiments were approved by the Ethics Committees of HVRI (Approval number: Heilongjiang-SYXK (Hei) 2017-009). Animals received appropriate care, and all experiments were conducted in accordance with the Principles of Laboratory Animals of the Ministry of Science and Technology of China.

### Animal immunization protocol and sample collection

Three outbred adult horses were randomly selected, and EIV seronegativity was confirmed in all three. The horses received intramuscular inoculation with two doses of the EIV inactivated vaccine (generated from A/equine/XinjiangFuyun/2007 (H3N8) (XJ07) strain) at 0 and 29 days. Serum samples were collected for the evaluation of the Hemagglutination inhibition (HI) titres on the indicated days (21,31,33,35,37,39,41,48,55,62 and 76 days). Peripheral blood mononuclear cells (PBMCs) were isolated from immune horses at the time point when the highest hemagglutination inhibition (HI) titre was observed using a Ficoll-Histopaque density gradient (density = 1.077 g/ml) for sorting HA-specific B cells.

### Sorting HA-specific B cells from EIV-inoculated equine PBMCs

Biotin–streptavidin conjugation was used to stain isolated equine PBMCs simultaneously with a bundle of monoclonal antibodies, including FITC anti-rat CD3 (Abcam, ab34722), PE-Cy7 anti-human CD21 (BD Biosciences, 561374), Aqua Dead Cell Stain Kit (Thermo Fisher, L-34965) and the EIV-HA-Y97F trimer labelled with BV421 (Biolegend, 405225) and APC (Thermo Fisher, 17-4317). A stepwise gating strategy was employed as follows: lymphocytes were gated based on FSC-A and SSC-A parameters; live cells were gated using aqua vital-AmCyan; B cells were gated with CD3^neg^CD21^pos^, and HA-bound B cells were gated using HA-Y97F mutant-trimer-BV421^pos^ conjugated HA-Y97F mutant-trimer-APC^pos^. The HA-Y97F mutant-trimer using a Foldon-guided self-assembly approach was engineered specifically to minimize nonspecific binding [17,18], as detailed in the supplementary methods. The flow-cytometric sorting was conducted in a BD FACS Aria III instrument. Single HA-baited B cells were directly sorted into 96-well PCR plate.

### Two-round nested PCR

The equine immunoglobulin gene primer pool was utilized to amplify equine immunoglobulin genes, and separate cDNA pools for heavy and light chains were constructed. Using a mixed cDNA pool, a two-round nested PCR process was sequentially performed. This aimed to amplify equine Ig genes (Fab) consisting of two variable domains (VH and VL), along with two constant regions (CH and CL), from heavy and light chains independently. In Round-1 of the nested PCR, pooled Tag-labelled equine leader sequences were employed as forward primers to amplify variable domains in either the heavy chains (VH) or the light chains (VL) separately. Reverse primers targeting non-variable regions in the equine sequences were used to amplify constant regions in either the heavy chains (CH) or the light chains (CL). Primers for the equine variable region in the light chain were used only for the amplification of the lambda chain (VL: λ), as the κ to λ ratio is 1:20 in equines [19,20].

For Round-2 of the nested PCR, the Tag was targeted to design forward primers for the subsequent nested amplification. Reverse primers specific to abbreviated equine constant CH1/CL1 genes with tails corresponding to homo constant gene IgG1 (CH) or Ig λ (CL) were designed aiming at facilitating recombination in the following step. Subsequently, cDNA fragments encompassing variable domains and constant regions within equine heavy chains or light chains were individually amplified from single memory B cells. All PCR products were identified using sequencing. The PCR products of the equine heavy chain genes were subsequently annotated with Complementarity-Determining Regions (CDRs) using the V-quest function in the IMGT information system and using the equine germline database as a reference [21]. Detailed primer sequences are provided in Supplementary Material (Tables 1–7).

#### Expression of recombinant Igs harbouring equine Ig (Fab)2 and homo Fc

All PCR products from candidate single B cells harbouring equine Fab genes were purified using a Qiagen PCR Purification kit (Qiagen, Valencia, CA). Subsequently, overlapping PCR products of the amplified equine Fab containing homo Fc in the heavy chains, as well as products containing Fc in the light chain from the same single equine B cell well, were co-transfected into HEK 293 T cells at a ratio of 1:1 using PolyFect (Qiagen, Valencia, CA). Co-transfected HEK 293 T cells were then cultured for the expression of recombinant immunoglobin.

### Screening recombinant Igs harbouring equine Ig (Fab)2 and homo Fc binding EIV-HA

We next assessed the ability of the recombinant immunoglobulins (mAbs) to bind to the HA protein [22]. We coated HA protein onto ELISA plates, blocked them, and then added purified mAbs from transfected HEK 293 T cell cultures. After incubation, we used a secondary antibody and measured binding using an ELISA reader. We determined the relative affinity of mAbs by calculating the effective concentration (EC_50_) required for half-maximal binding using GraphPad Prism. Positive mAbs were further processed for expression abundance in 293F cells. Recombinant antibodies were isolated, cultured, and purified using size-exclusion chromatography using a Superdex 200 Increase 10/300 GL column (GE Healthcare) as previously described [20,23,24]. The binding of purified mAbs to EIV-HA protein was confirmed using SPR assays. For further details, refer to the Supplementary Material.

### Screening of recombinant antibodies for neutralization

To identify the most effective recombinant antibodies capable of neutralizing various strains of equine influenza virus (EIV), virus neutralization assays were conducted. Influenza strains used in this assessment included a main circulating EIV strain of A/equine/XinjiangFuyun/2007 (H3N8) (XJ07), an avian origin equine IAV A/equine/Jilin/1989 (H3N8) (JL89), as well as two recently isolated strains (A/equine/GanSu/2022 (H3N8) (GS22) and A/equine/hulunbeier/1/2023 (H3N8) (HLBE23)) in our lab. The neutralization assay followed the guidelines outlined in the OIE Terrestrial Manual. Viral stocks were prepared in eggs, and the titre of the stock was determined by TCID_50_ assays using MDCK cells. Subsequently, 50 μl of serially diluted monoclonal antibodies (mAbs) or serum was mixed with an equal volume of culture medium containing 200 TCID_50_ virus and incubated for 1 h at 37 °C. The mixture was then transferred to MDCK cells in a 96-well plate and incubated for about 24 h at 37 °C. After removing the supernatant post-infection, each well received 100 μl of paraformaldehyde reagent for 10 min, followed by washing with PBS. Direct ELISA was then performed using anti-influenza A NP mouse monoclonal antibody. The negative control (VC) contained 200 TCID_50_ of EIV but without the antibodies, while the cell control (CC) contained only DMEM (with 1 μg/mL TPCK-trypsin). The neutralization efficiency of mAbs was calculated as previously described [25].

### In vivo validation of the screened candidate recombinant antibody

We tested equine recombinant mAbs as potential cross-reactive neutralizing antibodies. Mice were challenged with EIV to assess the efficacy of the antibodies in preventing and treating the infection. The EIV strain (A/equine/XinjiangFuyun/3/2007 (H3N8) (XJ07)) was adapted for mice, establishing a lethal challenge model with a dose of 6 × 10^7^ EID_50_. Forty BALB/c mice (aged 6–7 weeks) were randomly divided into groups (*n* = 10 per group). Two groups of challenged mice received a single intravenous dose of 20 mg/kg for either pre-exposure prophylaxis (PrEP) or therapeutic treatment (H81). One challenged group remained untreated, while another group was mock-challenged with PBS as a control. The mice were monitored daily, and body weight loss and deaths were recorded. On day 5, each from the challenged and medication groups (death), and on day 14, each from the PrEP, medication and sham groups, were euthanized. Lung samples were collected during autopsy for virus titration and histopathological analysis.

### Assessment of lung histopathological changes and viral titres

Autopsy was performed in randomly selected mice that had either died (in the challenged group and medication group) or that had undergone euthanasia (in other groups). Lung tissues were fixed in 4% paraformaldehyde, embedded in paraffin, and then sectioned and stained by the routine hematoxylin and eosin method (H&E) for histopathological examination. To determine the viral titre in lung tissue, the supernatant collected after tissue grinding was serially diluted by a factor of 10 and then inoculated into MDCK cells. Following virus absorption for 1 h, the virus was removed and MDCK cells were cultured in Opti-MEM supplemented with tosylsulfonyl phenylalanyl chloromethyl ketone (TPCK)-treated trypsin (1μg/mL). Cell supernatants were harvested at 24 h post-infection, and viral titres were determined by HA-specific assay.

### Paratope and epitope prediction of the screened recombinant equine Ig (Fab)2 and HA

To investigate how the screened recombinant equine Ig (Fab)2 binds to the HA antigenic epitopes, we used SWISS-MODEL homology modelling. Each Fab sequence was entered individually, along with its corresponding immunoglobulin variable domains in heavy and light chains, to predict the quaternary structures of both the antibody and the HA antigen. The prefusion state of HA (PDB ID: 7K37) served as the template for the modelling process. Two binding models were generated, one for trimeric HA and another for monomeric HA. The PyMOL software was utilized for structural analysis and figure preparation.

### Prediction of functional binding regions between the HA monomer and the potential equine Ig (Fab)2

To predict the paratope of potential HA neutralizing antibodies derived from recombinant equine Ig (Fab)2 and their corresponding antigenic epitopes, we employed homology modelling to predict the 3D structures of both the antibodies and HA. SWISS-MODEL was utilized with the prefusion state of HA (PDB ID: 7K37) as the template. The input included the sequences of the screened equine Ig (Fab)2 along with the corresponding immunoglobulin variable domains or constant regions. Molecular docking in Discovery Studio Visualizer (V4.5) was used to predict the most likely binding mode. Twenty theoretical models were constructed for statistical optimization based on minimum Probability Density Function (PDF) for Total Energy and minimum DOPE Score. The optimal complex model was used to predict the functional binding region between equine Ig (Fab)2 paratopes and EIV-HA antigenic epitopes. PyMOL was employed for structure analysis and figure preparation.

### Validation of each predicted epitope in EIV-HA ex vivo

Based on the predictive analysis conducted in PyMOL, 27 amino acid residues within the HA protein, which were identified as potentially influencing antibody binding, were selected for mutagenesis using a mutagenesis kit (New England Biolabs). Following this, expression plasmids containing HA mutants were constructed using the pCDNA3.3 vector. To evaluate the binding affinity of H81 to these mutants, both direct ELISA and SPR assays were conducted. By comparing the affinity kinetics before and after mutation, we identified the sites critical for antibody binding to HA. In the direct ELISA assay, microtiter plates were separately coated with HA mutant expression proteins, followed by the addition of H81 antibody, to assess their binding interaction. In the SPR assays, anti-EIV-HA antibodies were immobilized on the CM5 chip in HBS-EP buffer (10 mM HEPES pH 7.4, 150 mM NaCl, 3 mM EDTA, 0.005% surfactant P20). Binding analyses were conducted by injecting HA mutant protein solutions at various concentrations. Further details regarding the ELISA assay and SPR method can be found in the Supplementary Material.

### Statistical analyses

Treatment groups were compared using unpaired Student’s t-tests, two-way analysis of variance (ANOVA), or one-way ANOVA followed by Dunnett’s multiple comparisons test or Tukey’s multiple comparisons test, in the GraphPad Prism 7.0 software. Statistical parameters are indicated in the figures as follows: NS (not significant, *P *> 0.05), *P < 0.05, **P < 0.01, and ***P < 0.001.

## Results

### Generation and isolation of EIV-HA-specific single B cells

**Figure 1.**
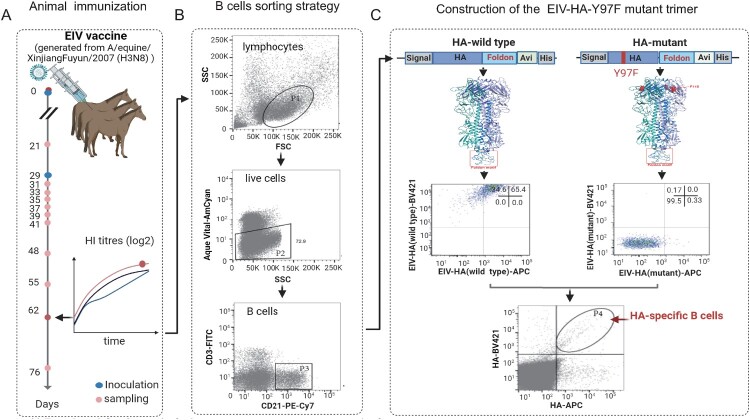
The protocol used to obtain equine single HA-specific B cells. (A) Three horses were vaccinated and received a booster dose of the equine influenza virus (EIV) inactive vaccine to induce a strong B cell immune response. EIV-HA-specific antibodies were measured using HI assays at specific time points. Peripheral blood mononuclear cells (PBMC) were isolated from the horse with the highest HI titre for single B cell sorting. (B) A stepwise gating strategy was employed to isolate single HA-specific B cells from PBMC. (C) Construction of the HA-Y97F mutant trimer to reduce nonspecific membrane binding.

To generate and isolate equine B cells that harboured antigen-bound B-cell receptors (BCRs) highly specific to HA, we implemented the efficient and cost-friendly workflow summarized in Figure 1(B). To enrich for EIV-HA-specific B cells, we employed the HA-Y97F mutant trimer as bait, which effectively enhanced specificity by eliminating SA-dependent nonspecific binding to host cell membranes. As shown in Figure 1(C), the HA trimer with the Y97F site mutation was chosen as the final gating strategy, resulting in a significant reduction in nonspecific membrane binding (65.4% vs 0.0%).

The horses were immunized as described above to elicit antigen-specific responses, and HI assay was conducted weekly prior to PBMC sorting (Figure 1(A)). To screen for neutralizing monoclonal antibodies (mAbs), PBMCs were selected from the immunized donor with the highest HI titre (1:256), reflecting the presence of robust circulating memory B cells producing potent neutralizing IgGs against EIV-HA. A total of one hundred thousand PBMCs underwent B cell isolation column purification to remove T cells (CD3^pos^ CD21^neg^ cells), resulting in four thousand B cells. These cells were subsequently stained with antibodies targeting cell surface markers and antigens, and sorted to isolate the CD3^neg^CD21^pos^ HA^pos^ population. Ultimately, 370 HA-specific B cells were sorted from 1 × 10^6^ PBMCs (Figure 1(C)).

### Stepwise amplification of equine F(ab) genes in single B cells

**Figure 2.**
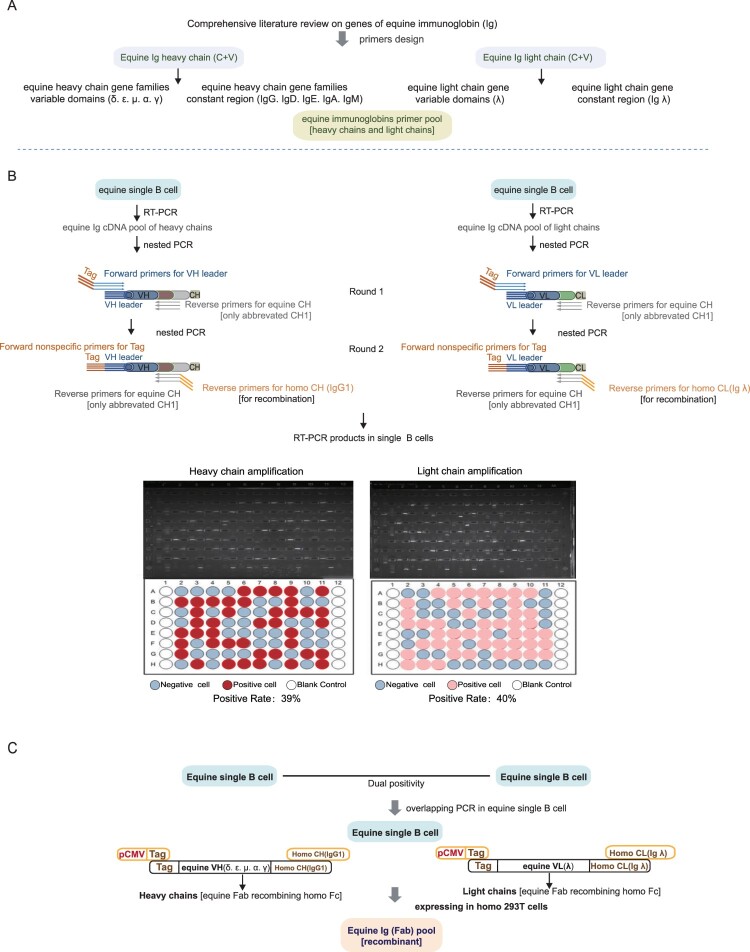
The stepwise protocol used to obtain equine monoclonal immunoglobin (Ig) antibodies from equine single B cells. (A) The equine Ig primer pool was constructed by isolating heavy chains and light chains separately, based on a comprehensive review of the literature. (B) The cDNA products of heavy chains and light chains of equine Ig (Fab) in single equine B cells were amplified using two-round nested PCR. (C) The equine Ig (Fab) was recombined with homo Ig (Fc) using overlapping PCR, enabling expression and purification in the homo system, and producing the recombinant equine Ig (Fab) pool.

In the two-round nested PCRs, 145 wells out of 370 harbouring single equine B cells tested positive for equine heavy chains (145/370, 39%), while 149 wells tested positive for the equine light (λ) chain (149/370, 40%). Of these, a total of 137 single equine B cells demonstrated dual positivity (137/370, 37%), expressing equine Ig genes (Fab with a short Fc-CH1 tail) from both heavy and light chains. These double positive B cells were selected for subsequent recombination (Figure 2(B)). Through overlapping PCR, equine Ig (Fab) genes from the heavy and light chains originating from the same single B cell were presumed to self-assemble into linear cassettes for expression and recombination with the homo constant region IgG1. These processes resulted in 137 recombinant monoclonal Igs containing equine Ig (Fab)2 and homo Ig (Fc) components derived from distinct single B cells (Figure 2(C)). The positivity rate observed in this screening platform is comparable to that seen in mouse and human single-B cell screening platforms, indicating that it is sufficient for subsequent screening aimed at identifying neutralizing antibodies.

### Expression and binding characteristics of recombinant antibodies

**Figure 3.**
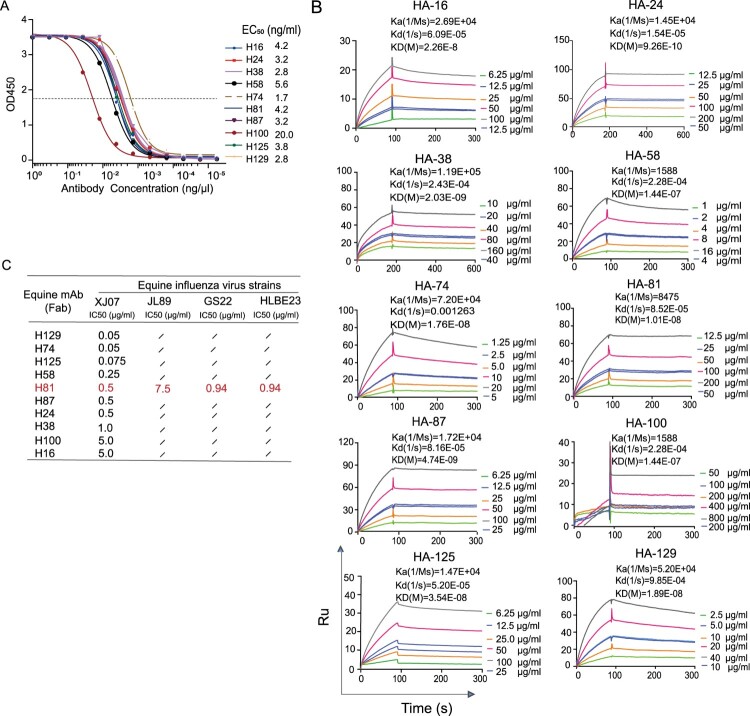
Stepwise validation of screened equine monoclonal Igs (Fab) against EIV-HA. (A) Ten equine Ig (Fab) antibodies were evaluated through ELISA binding assays against EIV-HA to calculate the EC_50_ value. (B) The binding affinities of these antibodies to EIV-HA were assessed using surface plasmon resonance (SPR). (C) The abilities of ten equine recombinant antibodies to neutralize the main prevalent EIV strain XJ07, GS22, HLBE23 and JL89 (an avian-like equine influenza virus) were investigated.

A total of 137 recombinant Igs, including equine Ig (Fab)2 and homologous Ig (Fc), were individually expressed in co-transfected HEK 293 T cells, resulting in the generation of 23 recombinant equine antibodies through random pairing. To verify binding specificity, we initially conducted ELISA assays on these antibodies. As anticipated, all 23 recombinant equine Ig (Fab)2 and homo Fc antibodies showed binding to EIV HA with EC_50_ values < 100 ng/mL. The 10 antibodies with the highest binding affinities (EC_50_ < 20 ng/mL) were further characterized using SPR assays (Figure 3(A)). Consistent with the ELISA results, all 10 antibodies demonstrated robust binding signals to EIV-HA. SPR assays further revealed dissociation constants (KD) ranging from 10^−8^ to 10^−10^ M for these antibodies, as depicted in Figure 3(B). Interestingly, a consistent pattern in the affinity levels of antibodies was observed across both methods. For example, the H100 antibody consistently exhibited the lowest affinity. Taken together, our findings suggest that in our screened equine Ig (Fab)2 library, ten recombinant Igs specifically bind to EIV-HA with high binding affinities.

To characterize the function of the isolated recombinant mAbs, we further analyzed their neutralization capacity in MDCK cells using a panel of EIV strains. The micro-neutralization assay revealed that ten recombinant mAbs efficiently neutralized the original parent strain (XJ07) at concentrations ranging from 0.05 μg/mL to 5.0 μg/mL. Specifically, H81 exhibited neutralizing activity against the virulent EIV strain (JL89) with an IC_50_ value of 7.5 μg/mL, indicating its high virus-neutralizing potency (Figure 3(C)). This observation was corroborated across additional EIV strains, where H81 effectively protected against infection by other A/H3N8 EIV viruses (GS22 and HLBE23) with IC_50_ values (0.94 and 0.94 μg/mL). Consequently, H81, identified as cross-reactive, was selected as the candidate therapeutic mAb for subsequent in silico prediction and *in vivo* validation.

### Validation of the recombinant antibodies in EIV-challenged mice

**Figure 4.**
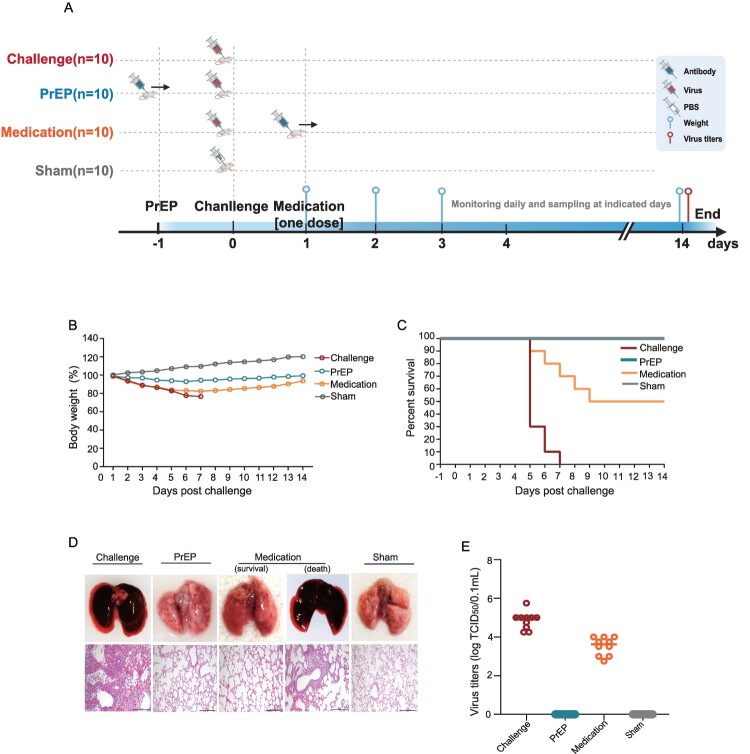
*In vivo* validation of the screened equine Ig Fab (H81) against an adapted EIV (H3N8) in lethally challenged mice. (A) The timeline of the *in vivo* experiment involved forty mice that were divided into groups receiving medication (one-dose H81), PrEP (one-dose H81), challenge (adapted H3N8), and sham challenge. Weight loss (B) and survival curves (C) were measured simultaneously across four groups. (D) Both overall and pathological examinations were conducted across the four groups. (E) The viral titres in lung distension were simultaneously assessed using the TCID_50_ assay.

Four groups of mice were monitored and compared (the timeline is shown in Figure 4(A)). Compared with the lethally challenged control group, both the therapeutic group and the prophylactic group (PrEP) showed milder clinical symptoms, as indicated by insignificant weight loss (Figure 4(B)). Additionally, deaths occurred in both the medication group and the challenged group: specifically, five deaths were observed in the one-dose H81 medication group. In contrast, the lethally challenged control group experienced 100% mortality by 8 days post-infection (*dpi*) (Figure 4(C)). In contrast, mice that underwent PrEP showed 100% protection against EIV challenge, with only mild nonspecific changes such as slight edema and congestion observed via histopathological microscopy (Figure 4(D)). Importantly, viral titres in autopsied mice consistently showed undetectable levels of virus in lung tissues from the PrEP group, in stark contrast to markedly high viral titres observed in the lethal challenge group (TCID_50_/0.1 mL:10 (4.25)−10 (5.75)) (Figure 4(E)). Despite the therapeutic group showing a 50% protection rate, the virus titres in the mice were significantly lower compared to those in the challenged control group (10 (3.52) *vs* 10 (4.85)). These data indicate that the H81 recombinant antibody has potential for protecting and treating EIV infections *in vivo*.

### Prediction of the functional binding region between the screened recombinant equine Ig (Fab)2 and EIV-HA

**Figure 5.**
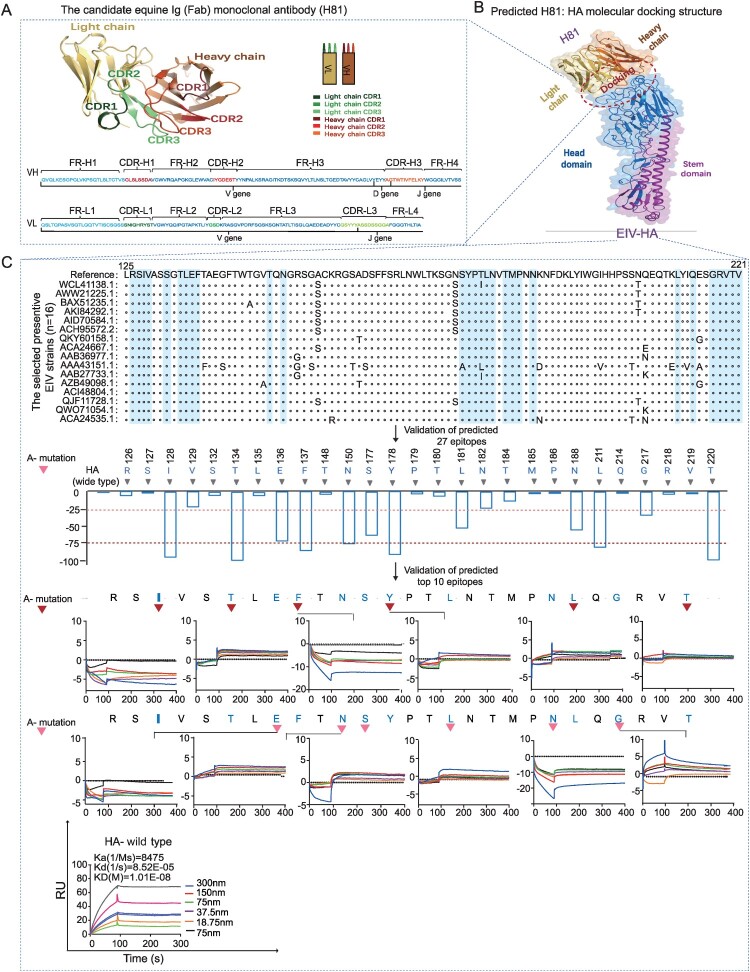
The molecular docking prediction and validation between paratopes on the screened equine Ig Fab (H81), as well as epitopes on HA derived from EIV (H3N8). (A) The anticipated crystal structure featuring complementarity-determining regions (CDRs) located on the screened equine Ig (Fab) H81, which targets HA-EIV. (B) The projected molecular docking site where paratopes on the screened equine Ig (Fab) H81 interact with epitopes on HA-EIV H3N8. A total of 27 dispersed amino acid residues were identified as spanning the established receptor binding site of HA in EIV. (C) A sequence alignment was conducted on the HA protein's 27 anticipated epitopes using 16 selected representative EIV strains, as identified through sequence analysis. The 27 predicted residues on HA were subjected to A-mutagenesis, and binding validation via an ELISA assay was conducted (middle). The binding affinities of the top 12 ELISA-validated amino acid residues were then further evaluated using an SPR assay (lower).

To map the binding epitopes of the mAb to HA, we sequenced the candidate recombinant equine Ig (Fab)2 (H81). The amino acid residues of the screened recombinant equine Ig (Fab)2, and its predicted crystal structure are shown in Figure 5(A) (left). Three CDRs and four FRs were predicted in both the heavy and light chains. Predicted Ig gene families and their corresponding gene loci were also identified. Additionally, the predicted epitope domain of H81 largely overlapped with the receptor binding site of EIV-HA H3N8, as revealed by X-ray crystallography [26]. The predicted epitope contained 27 amino acids in the H81 monomer (Figure 5(A), right). Mutations in EIV-HA reduced binding to H81, particularly in 12 amino acids, including positions 128,134,136,137,150,177,178,181,188,211,217 and 220 (cut off value = −25 via ELISA). These results were further validated using affinity kinetics (Figure 5(C)). It was shown that the affinity kinetics of these 12 A-mutated amino acids were greatly reduced to approximately 0 Ru (kinetics ranged from −5 Ru to +8 Ru) compared to the wild strain (Kinetics = 80 Ru). This result indicates that these 12 amino acids are crucial for the binding of H81 to HA. Subsequently, we downloaded all 301 available full-length HA sequences of equine influenza virus strains (H3N8) from the Influenza Virus Database of NCBI (https://www.ncbi.nlm.nih.gov/genomes/FLU/Database) and GISAID (https://www.gisaid.org). Using a neighbor-joining phylogenetic tree, we carefully selected representative strains (16 in total) from each branch. We identified 12 amino acid sites within the HA sequence associated with strains neutralized by H81 and are relatively conserved across strains, except for a single-site mutation observed in strain AAA43151.1 (Figure 5(B) and Supplement Figure S1). To further validate our results, we conducted additional comparisons of the HA sequences from four strains neutralized by H81, focusing on identifying the key binding sites of the H81 antibody. Among the 12 key sites identified in this study, strain JL89 contains 9 of these sites, excluding positions 137, 150, and 177. In contrast, strains GS22 and HLBE23 include all sites except position 181. These variations in binding site composition may contribute to the observed weaker neutralization ability of H81 against JL89 compared to other strains (Figure 3(C)). Collectively, these findings indicate that the predicted epitopes on EIV-HA (H3N8) are functional and have consistent sequences.

## Discussion

**Figure 6.**
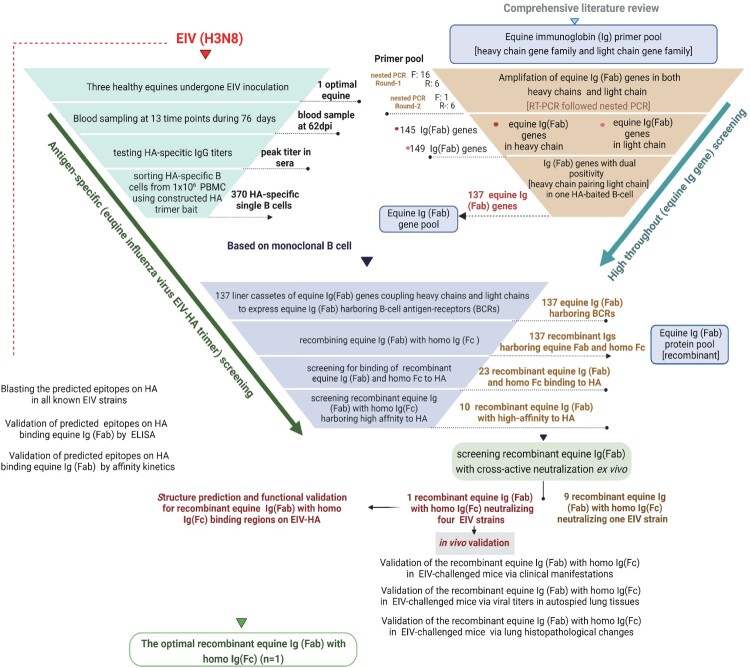
A stepwise protocol of the screening-and-validating platform presented here. The left triangle outlines the protocol for screening HA-specific antibodies in individual B cells obtained from the equine host under study. The right triangle details the process for generating equine Ig (Fab). The lower portion of the diagram illustrates cross-reactive neutralization, subsequent *in vivo* validation, and in silico prediction. This platform was implemented to leverage both antigen specificity and antibody diversity within single B cells.

Using equine influenza as a model, we constructed a rapid platform for the screening of EIV-HA-targeted cross-neutralizing antibodies based on the human BCR single B-cell screening approach [27,28]. This platform was developed using four major steps. Firstly, we screened for HA-specific memory B cells from the host with the highest titre of anti-HA IgG. Secondly, we performed high-throughput rearrangement and reassembling analysis on 370 equine memory B cells within the equine Ig gene family pool. Subsequently, two rounds of nested PCR were conducted to selectively amplify the equine Ig (Fab) genes, followed by recombination of the equine Ig (Fab)2 with homo Ig (Fc) using overlapping PCR. Furthermore, we conducted two assays to screen for recombinant antibodies with high binding affinity to EIV-HA. Finally, the purified recombinant antibodies were evaluated for their neutralization activity *ex vivo*. One mAb, named H81, was successfully screened against four EIV strains using the developed platform. Subsequently, H81 underwent *in vivo* validation both clinically and pathologically, as well as in silico prediction. The overall stepwise platform is illustrated schematically in Figure 6.

Throughout the stepwise process of monoclonal antibody screening, the antigenic specificity, exemplified by HA, was naturally tracked from host inoculation to HA-gated B-cell sorting and further to HA-specific equine Ig (Fab) screening, facilitated by a high-throughput platform employing equine Ig primer pools. Following this, the identified cross-neutralizing mAb was presumed to have inherent selective potential against ongoing EIV antigenic drift/shift. HA, through its rapidly mutating binding receptor, attaches to host cells, facilitating continuous immunoevasion. Therefore, it is strategically imperative for host immunity mAbs to obstruct virus receptor binding sites while evading “exposure” as mutation targets. Nonetheless, screening highly conserved epitopes within the globular HA head, where mutations persist, poses considerable challenges [29].

Using the current platform, we successfully identified 12 amino acid residues distributed within and surrounding the receptor binding site of the membrane-distal HA head. These 12 predicted amino acid residues were found to be significantly conserved across the main circulating EIV strains. Feedback analysis was conducted on 12 key amino acids across four strains, including the main circulating strain of equine influenza (XJ07), two newly isolated virus strains, and an avian-like equine influenza virus. This analysis further validates the broad spectrum of antibodies associated with these key amino acids.

This version maintains clarity and coherence while incorporating the information about the antibodies related to the amino acids analyzed.

Subsequent *ex vivo* validation underscored the indispensable function of the predicted binding domain in facilitating interaction between the H81 antibody and the EIV-HA head. Particularly noteworthy were two epitopes (150Asn and 211Leu), positioned at the periphery of the HA receptor binding site, which emerged as pivotal in dual *ex vivo* assays. Consequently, H81, with its cross-neutralizing potential, emerges as a promising candidate antibody in combating EIV antigenic shift/drift.

The majority of vaccines against human influenza viruses target the receptor binding site on HA. However, despite this, it is not easy to sustain functionality in these vaccines, as the virus may selectively expose its antigen as a “bait” to launch immunoevasion or hide its antigen to exhaust “invalid” BCR reactivation, together leading to antibody/vaccine resistance [17,29–31]. on our present findings from the rapid screening-and-validating platform, we cautiously suppose that an exposure-and-bait strategy from HA antigens would address a balance between delicate immunoevasion and always last-generation robust antibodies. Using the present platform, which simulates this natural contest between the virus and its host immunity, only one equine monoclonal (Fab)2 antibody demonstrated resistance to viral immunoevasion due to antigenic drift/shift: this might represent a winner of the counter-bait strategy. Upon characterization of H81, we observed that it exhibited moderate binding affinity to its parental strain, compared to the other three mAbs, which displayed high affinity and predicted epitopes that did not obstruct the equine receptor binding site (Supplementary Figure S2). Low-affinity molecular binding interactions have also been successfully screened using computational design methods based on the conserved surface patch on the stem of HA [32]. The observation that numerous vaccines generating robust neutralizing antibodies against influenza and HIV-1 have struggled to keep pace with ongoing viral evolution underscores the importance of developing resistance against viral immunoevasion as a critical feature in vaccine design. While the arousal of a robust immune response is undoubtedly crucial, it is equally essential for vaccines to effectively counteract the strategies employed by viruses to evade immune detection and neutralization.

In this study, we identified a single cross-neutralizing monoclonal antibody (mAb) that demonstrated potential resistance to different EIV-HAs. This resistance was attributed to the mAb's coverage of the conserved domain of the antigenic “bait” and its moderate binding affinity for the binding “bait.” Through crystal structure prediction of the epitope-paratope docking model, we propose that such selective adaptation by the host B cell receptor (BCR) could influence the dynamic interplay between the virus and host immunity, providing a benefit to the host without being readily “detected” by a virus that undergoes variable changes over time. This selective adaptation mechanism highlights a potential avenue for developing vaccines that can better withstand viral evolution and immunoevasion tactics [26,33]. This platform provides an alternative approach to the development of pan-influenza vaccines by rapidly pinpointing interactive domains between the virus and its host. The rapid identification of these crucial interaction points offers substantial advantages for disease therapy and vaccine development, especially in response to emerging viruses. Specifically, the platform's ability to swiftly identify optimal antigen-specific monoclonal antibodies (BCRs) is particularly valuable in cases where host or intermittent host Ig gene families are limited. Here we identified five equine Ig gene families in heavy chains, three fewer than the eight gene families subsequently published. Utilizing pooled RT–PCR products containing five leader sequences, we isolated 137 monoclonal B cells that are hypothesized to encompass the majority of equine BCRs. The identification of two equine Ig gene families within the light chains led us to select the predominant family (λ) for constructing the equine Fab VH-VL cassette. The successful screening of H81 underscored the notable efficacy of our current screening platform. Furthermore, the rapid and interactive simulation of virus natural selection and host BCR adaptation provided valuable insights into the dynamic interaction between viruses (epitopes) and hosts (paratopes) [34].

Our findings based on this platform suggest that host BCR adaptation may result in paratopes characterized by a blocking receptor binding site, encompassing dispersed conserved sequences, and exhibiting low-to-moderate affinity, which could be considered an optimal outcome. This highlights the potential of our platform to inform the design of therapeutics and vaccines that target crucial interaction points between viruses and hosts, ultimately improving our ability to combat infectious diseases.

## Supplementary Material

Supplemental Material

S1.eps

TableS4.docx

Supplementary..pdf

TableS1.docx

s2.eps

TableS2.docx

TableS6.docx

TableS5.docx
