# Supplementary material for: A platform for the rapid screening of equine immunoglobins F (ab)2 derived from single equine memory B cells able to cross-neutralize to influenza virus
Source: Emerg Microbes Infect. 2024 Sep 27;13(1):2396864. doi: 10.1080/22221751.2024.2396864 (PMC11441081; doi:10.1080/22221751.2024.2396864)
Supplement: Supplemental Material [file TEMI_A_2396864_SM5082.docx]

Table S3 Equine Ig light chain (κ) Primers for Round-1 nested PCR

| Direction | PRIMER ID | 5’-3’ SEQUENCE |
| --- | --- | --- |
| Forward | V_κ_1-1 | ATCGACGTTGGACTCCAGAGATGAGGKTCYCTGCTCAGCTCCT |
|  | V_κ_1-2 | ATCGACGTTGGACTCCAGAGATGAAATTCSYTRSTCAGCTCCT |
|  | V_κ_1-3 | ATCGACGTTGGACTCCAGAGATGAGCTTCCAGGCCCAGCTC |
|  | V_κ_1-4 | ATCGACGTTGGACTCCAGAGATGATGTYGCWGACAMAGKTCCTT |
|  | V_κ_1-5 | ATCGACGTTGGACTCCAGAGATGATGTSRSAGACACAGGTCCT |
|  | V_κ_1-6 | ATCGACGTTGGACTCCAGAGATGGGCTCCCAGGCTCAGCTC |
|  | V_κ_1-7 | ATCGACGTTGGACTCCAGAGATGGTGTCCCCATCACAGCTC |
|  | V_κ_1-8 | ATCGACGTTGGACTCCAGAGATGAGGYTCCCTGCTCAGCTCCT |
|  | V_κ_1-9 | ATCGACGTTGGACTCCAGAGATGGGGTCAAGGGCTTCACTG |
|  | V_κ_1-10 | ATCGACGTTGGACTCCAGAGATGATGTCATGGACTCAGATCCT |
| Reverse | C_κ_-CR1 | GAA GCC ATA CAC CAA GCA TAC GAC AGA |
