# Supplementary material for: A platform for the rapid screening of equine immunoglobins F (ab)2 derived from single equine memory B cells able to cross-neutralize to influenza virus
Source: Emerg Microbes Infect. 2024 Sep 27;13(1):2396864. doi: 10.1080/22221751.2024.2396864 (PMC11441081; doi:10.1080/22221751.2024.2396864)
Supplement: TableS4.docx [file TEMI_A_2396864_SM6151.docx]

Table S4 Primers of equine Ig heavy chains in constant regions for Round-2 nested PCR

| Direction | PRIMER ID | 5’-3’ SEQUENCE |
| --- | --- | --- |
| Forward | V_H_-int | ATCGACGTTGGACTCCAGAG |
| Reverse | IgG-R2 | CACCGGTTCGGGGAAGTAGTCCTTGACMAGGCAGCCCASGGSMACCGTGG |
|  | IgE-R2 | CACCGGTTCGGGGAAGTAGTCCTTGACCAGGCAGCCCAGTGTGATGTTAG |
|  | IgA-R2 | CACCGGTTCGGGGAAGTAGTCCTTGCCCTTGATCAGGCAGGCGATGACCA |
|  | IgD-R2 | CACCGGTTCGGGGAAGTAGTCCTTGACCAAGCAGGCCAGGCTCACAACGT |
|  | IgM-R2 | CACCGGTTCGGGGAAGTAGTCCTTGGCTAGGCAGCCCACAGCCACCAGGC |
