# Supplementary material for: A platform for the rapid screening of equine immunoglobins F (ab)2 derived from single equine memory B cells able to cross-neutralize to influenza virus
Source: Emerg Microbes Infect. 2024 Sep 27;13(1):2396864. doi: 10.1080/22221751.2024.2396864 (PMC11441081; doi:10.1080/22221751.2024.2396864)
Supplement: Supplementary..pdf [file TEMI_A_2396864_SM6150.pdf]

Figure S1

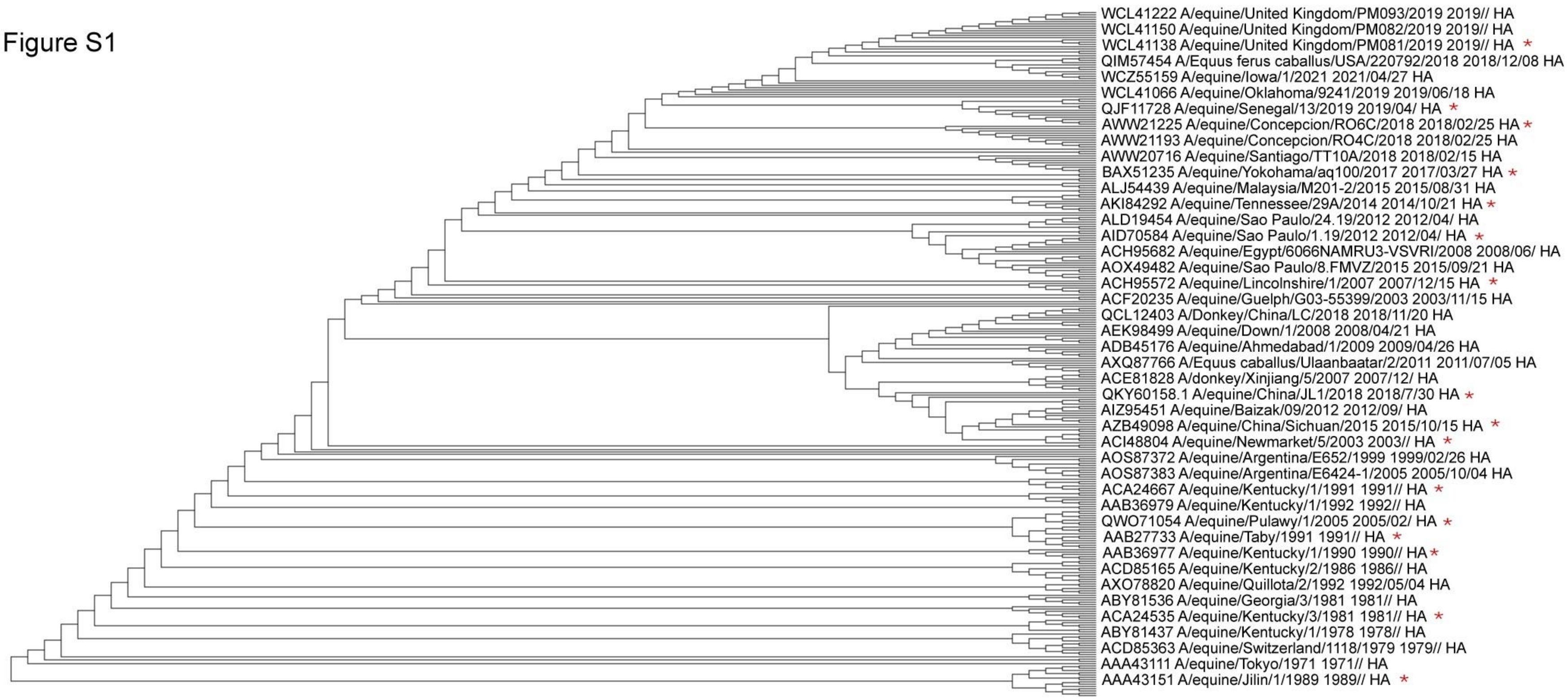

Supplementary Figure S1. Construct a neighbor-joining phylogenetic tree using n=301 complete equine influenza A (H3N8) hemagglutinin (HA) gene sequences downloaded from the Influenza Virus Database of NCBI (https://www.ncbi.nlm.nih.gov/genomes/FLU/Database) and GISAID (https://www.gisaid.org). Highlight selected representative strains with a red start shape.

Figure S2

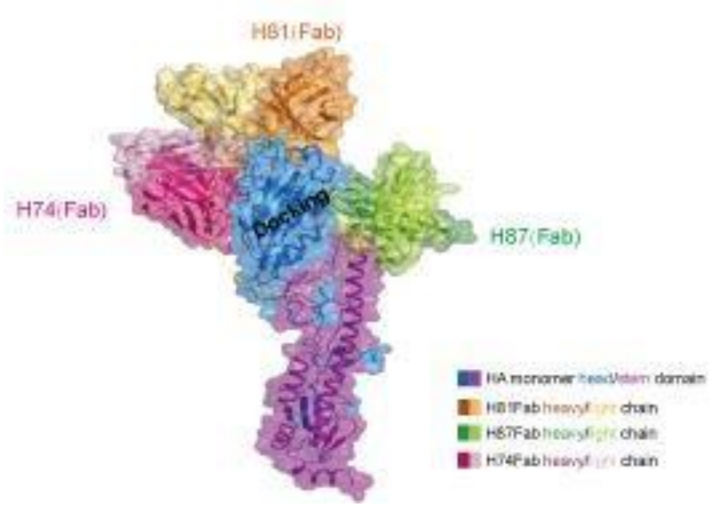

Supplementary Figure S2. Construct a neighbor-joining phylogenetic tree using n=301 complete equine influenza A (H3N8) hemagglutinin (HA) gene sequences downloaded from the Influenza Virus Database of NCBI (https://www.ncbi.nlm.nih.gov/genomes/FLU/Database) and GISAID (https://www.gisaid.org). Highlight selected representative strains with a red start shape.

Supplementary Material

Construction of an equine immunoglobin gene primer pool

To the best of our knowledge, no equine immunoglobin genes with corresponding gene loci were available at the start of the experiment. Based on a comprehensive literature review [20], equine Ig gene families with supposed variable domains and constant regions either in the heavy chains or in the light chains were predicted with reference to the homo immunoglobin database. A comprehensive literature review enabled us to design a primer pool for amplifying both the supposed five equine heavy chains (VH: γ, δ, α, μ and ε) and the two light chains (VL: λ, κ) in the variable domains, as well as the five heavy chains (CH: IgG, IgE, IgA, IgD, IgM) and two light chains (CL: Ig λ, Ig κ) in the constant regions.

Construction of the EIV-HA trimer and EIV-HA-Y97F mutant trimer

An EIV-HA trimer was constructed to bait HA-baited B cells, and an EIV-HA-Y97F mutant trimer was also generated to assess enhanced gating specificity in equine PBMCs. Trimerized HA, including HA1 (head domain) and HA2 (stem domain), from an isolate of the main EIV strain circulating in China (A/equine/XinjiangFuyun/3/2007 (H3N8) (XJ07)), was constructed using a Foldon-guided self-assembly approach. The extracellular domain of HA was cloned into a plasmid containing a signal peptide and a hexa-histidine affinity tag at the C-terminus for expression in HEK 293T cells. A Y97F mutant of HA was constructed as a gating reference, to reduce the nonspecific binding [17].

ELISA assay

To analyze the reactivity of recombinant antibodies to EIV-HA, 96-well ELISA plates were coated overnight at 4 °C with 0.2 μg/mL of recombinant HA protein. Following blocking with 1 % (w/v) BSA in PBST, purified recombinant antibodies (10 μg/mL) were added to the wells and incubated for 1 hour at room temperature (RT), followed by incubation with an HRPconjugated anti-mouse IgG antibody (1:2000) for 1 hour at RT. The enzyme signals were detected using the HRP substrate 3,3',5,5'-tetramethylbenzidine (TMB: BD Bioscience, San Jose, CA, USA), and the reaction was terminated with 2.5 M H_2SO_4.The absorbance at 450 nm was measured using a microplate reader (BioTek, USA).

Surface Plasmon Resonance Assays

The kinetic affinity of each purified recombinant equine Ig (Fab)2 to the EIV-HA trimer was assessed using SRP with the Biacore×100 System (GE Healthcare), following a previously described method [22]. An anti-homo Fc CM5-chip was prepared by covalently coupling with a human antibody capture kit (GE Healthcare, BR100839). Anti-EIV-HA antibody ligands were then immobilized as reference standards on the CM5 chip. EIV-HA protein samples were injected into the experimental channel at varying concentrations at a flow rate of 40 μl/min for a binding time of 90 seconds and a dissociation time of 5-10 minutes. Chip surfaces were regenerated after each cycle using 10 mM glycine at pH 1.5, with a flow rate of 30 μl/min and a regeneration time of 90 seconds. The kinetics were analyzed using Biacore×100 Evaluation Software (version: 2.0.1). The kinetic rate constants (K-on and K-off) were modeled to determine the affinity kinetic dissociation constant (K_D).
