# Supplementary material for: A platform for the rapid screening of equine immunoglobins F (ab)2 derived from single equine memory B cells able to cross-neutralize to influenza virus
Source: Emerg Microbes Infect. 2024 Sep 27;13(1):2396864. doi: 10.1080/22221751.2024.2396864 (PMC11441081; doi:10.1080/22221751.2024.2396864)
Supplement: TableS1.docx [file TEMI_A_2396864_SM6149.docx]

Table S1 Equine Ig heavy chains Primers for Round-1 nested PCR

| Direction | PRIMER ID | 5’-3’ SEQUENCE |
| --- | --- | --- |
| Forward | V_H_1-1 | ATCGACGTTGGACTCCAGAGATGGRCTGGAGCTGGAGMATCCTCT |
|  | V_H_1-2 | ATCGACGTTGGACTCCAGAGATGGACACACTGTATCCCACCCTC |
|  | V_H_1-3 | ATCGACGTTGGACTCCAGAGATGRAGTTTGGRCTGAKMTGGRYTTT |
|  | V_H_1-4 | ATCGACGTTGGACTCCAGAGATGAGRMKSYTGKGTCTTCTCCTTT |
|  | V_H_1-5 | ATCGACGTTGGACTCCAGAGATGARTCACCTGTGGTTCTTCCTC |
|  | V_H_1-6 | ATCGACGTTGGACTCCAGAGATGGCCCCTCTCCTGGTCATCTTC |
|  | V_H_1-7 | ATCGACGTTGGACTCCAGAGATGGGCTCTGCCACTGAACTT |
| Reverse | IgG-CR1 | AGTTCCAGGACACBKTCACYGGCTC |
|  | IgE-CR1 | GGTCACGGTCACTGGCTCCGGGAAGTA |
|  | IgA-CR1 | CTTCACGGGCTCTGGGAAGCCCAGCGG |
|  | IgD-CR1 | AGTGACCTGCACCGGCTCTGGGAAGTA |
|  | IgM-CR1 | CAAGGAGAAGGTGATGACGTTGGGTAG |
