# Supplementary material for: A platform for the rapid screening of equine immunoglobins F (ab)2 derived from single equine memory B cells able to cross-neutralize to influenza virus
Source: Emerg Microbes Infect. 2024 Sep 27;13(1):2396864. doi: 10.1080/22221751.2024.2396864 (PMC11441081; doi:10.1080/22221751.2024.2396864)
Supplement: TableS6.docx [file TEMI_A_2396864_SM6146.docx]

Table S6 Primers of equine Ig light chain(κ) in constant regions for Round-2 nested PCR

| Direction | PRIMER ID | 5’-3’ SEQUENCE |
| --- | --- | --- |
| Forward | Cκ-int | ATCGACGTTGGACTCCAGAG |
| Reverse | Cκ-R2 | CAGCAGGCACACAACAGAGGCACTTCCACTGCTTAACTCCTCAGA |
