# Supplementary material for: A platform for the rapid screening of equine immunoglobins F (ab)2 derived from single equine memory B cells able to cross-neutralize to influenza virus
Source: Emerg Microbes Infect. 2024 Sep 27;13(1):2396864. doi: 10.1080/22221751.2024.2396864 (PMC11441081; doi:10.1080/22221751.2024.2396864)
Supplement: Supplementary file 7 [file TEMI_A_2396864_SM6147.docx]

Table S2 Primers of equine Ig light chain (λ) for Round-1 nested PCR

Direction	PRIMER ID	5'-3' SEQUENCE
Forward	V_{λ}1-1	ATCGACGTTGGACTCCAGAGATGGCCTGGWSCCSKCTCCTYCTCRCC
	V_{λ}1-2	ATCGACGTTGGACTCCAGAGATGRCCTGGRCTCTGYTCCT
	V_{λ}1-3	ATCGACGTTGGACTCCAGAGATGGCCTGGACMCYTCTCCT
	V_{λ}1-4	ATCGACGTTGGACTCCAGAGATGKCCTGKACTCCTCTCMTCCTC
	V_{λ}1-5	ATCGACGTTGGACTCCAGAGATGGCCTGGGTGCCACTCCTGCTC
	V_{λ}1-6	ATCGACGTTGGACTCCAGAGATGGCCTGGAYRGTGCTTCTTCTC
	V_{λ}1-7	ATCGACGTTGGACTCCAGAGATGGCCTGGGCTCCACTCATCCTC
	V_{λ}1-8	ATCGACGTTGGACTCCAGAGATGGCCTGGWCCCCTCTCCTCCACTCCTGCT
	V_{λ}1-9	ATCGACGTTGGACTCCAGAGATGGCCTGGACCCCTCTCTT
Reverse	C_{λ}-CR1	AGGTAGCTGCTRGCCGCGTACTTGCC
